# Clinical course and management challenges in Lafora disease: a narrative analysis in an Apulian cohort

**DOI:** 10.1186/s13023-025-03976-x

**Published:** 2025-08-21

**Authors:** Giuseppe d’Orsi, Maria Teresa Di Claudio, Antonella Liantonio, Paola Imbrici, Cosimo Damiano Altomare, Orazio Palumbo, Pietro Palumbo, Mario Benvenuto, Nicola Gambacorta, Graziano Lolli, DEFEAT-LD study group, Francesca Bisulli, Cinzia Costa, Giuseppe Damante, Lidia Di Vito, Valentina Imperatore, Laura Licchetta, Raffaele Lodi, Lorenzo Muccioli, Paola Mantuano, Serena Mazzone, Roberto Michelucci, Elena Pasini, Paolo Prontera, Maria Tappatà, Luca Vignatelli, Corrado Zenesini, Massimo Carella

**Affiliations:** 1 Neurology Unit, Fondazione IRCCS Casa Sollievo della Sofferenza, Viale Cappuccini, 1, 71013 San Giovanni Rotondo, FG Italy; 2 Department of Pharmacy - Drug Sciences, University of Bari “Aldo Moro”, Bari, Italy; 3 Division of Medical Genetics, Fondazione IRCCS Casa Sollievo della Sofferenza, San Giovanni Rotondo, FG Italy; 4 Department of Cellular, Computational and Integrative Biology - CIBIO, University of Trento, Trento, Italy; 5 Full Member of the European Reference Network for Rare and Complex Epilepsies (EpiCARE), IRCCS Istituto Delle Scienze Neurologiche di Bologna, Bologna, Italy; 6 Department of Medicine and Surgery, University of Perugia, Perugia, Italy; 7 Section of Neurology, S. Maria Della Misericordia Hospital, Perugia, Italy; 8 Department of Medicine (DAME), University of Udine, Udine, Italy; 9 Institute of Medical Genetics, Udine University Hospital, Udine, Italy; 10 Department of Medicine and Surgery, University of Perugia, Perugia, Italy; 11 Medical Genetics Unit, S. Maria Della Misericordia Hospital, Perugia, Italy; 12 Dipartimento di Farmacia- Scienze del Farmaco, Università degli studi di Bari Aldo Moro, Bari, Italy

**Keywords:** Lafora disease, Late stage, Status epilepticus, Medical complications, Management, Electroclinical features

## Abstract

**Background:**

Lafora disease (LD) is an ultra-rare, autosomal recessive neurodegenerative disorder characterized by the accumulation of Lafora bodies in the brain, leading to drug-resistant epilepsy, myoclonus, progressive dementia, and cerebellar dysfunction. This retrospective study describes the clinical course and management challenges of LD in a cohort of patients from the Apulia region of Southern Italy, where the disease prevalence appears to be higher than in other populations.

**Methods:**

We retrospectively analyzed clinical, electroencephalographic, and management data from six unrelated families with a confirmed diagnosis of LD, followed at the Neurology Unit of the Scientific Institute Casa Sollievo della Sofferenza Hospital between 2010 and 2024. Demographic information, clinical presentation, treatment history, disease progression, and outcomes were collected.

**Results:**

Our analysis identified three distinct electroclinical stages: an initial Presenting Symptoms Stage with the onset of seizures and subsequent development of myoclonus; a Progressive Neurodegeneration Stage characterized by drug-resistant epilepsy, dementia, and ataxia; and a Terminal Stage marked by severe disability, frequent seizure emergencies, and medical complications. Management in the late stages proved particularly challenging, requiring a multidisciplinary approach to address refractory seizures, status epilepticus, and medical complications such as aspiration pneumonia and respiratory failure. Home-based care, with specialized team support, played a crucial role in minimizing hospitalizations.

**Discussion:**

Our findings underscore the importance of early diagnosis and a multidisciplinary approach in the management of LD. The late stages of the disease are characterized by significant clinical challenges necessitating close collaboration among neurologists, epileptologists, and other healthcare professionals, supported by effective home-based care. The apparent higher prevalence in Apulia warrants further investigation into potential genetic or environmental factors.

**Conclusion:**

This study highlights the significant clinical burden of LD and emphasizes the importance of multidisciplinary management, particularly in the advanced stages. Home-based care supported by specialized teams and caregivers is essential for optimizing patient well-being. Further research is needed to identify early biomarkers and develop targeted therapies for this devastating condition.

## Introduction

Lafora disease (LD, OMIM# 254780), a rare, autosomal recessive neurodegenerative disorder, affects approximately 300 individuals worldwide and is characterized by the accumulation of insoluble polyglucosan deposits (Lafora bodies) in the brain due to mutations in the *EPM2A* or *NHLRC1* genes [1]. The primary clinical manifestations of LD include drug-resistant epilepsy, refractory status epilepticus, myoclonus, progressive dementia, and cerebellar dysfunction, often leading to death within a decade of symptom onset [1–3]. Despite significant advancements in our understanding of the underlying molecular mechanisms, there are currently no effective disease-modifying therapies available for LD. This unmet medical need underscores the urgency of developing novel treatment approaches. This study focuses on a cohort of patients with LD from the Apulia region of Southern Italy, where the incidence of the disease appears to be higher than in other populations [4]. The reasons for this increased prevalence are not fully understood but may be related to genetic founder effects or specific geographical or cultural factors that have historically influenced the population structure of this region. The Italian national registry could provide valuable insights to address this question and facilitate genetic counseling. Previous studies have described the clinical course of LD, including the emergence of drug-resistant epilepsy, progressive neurodegeneration, and ultimately, death [1, 3, 5–11]. However, limited data exist on the long-term management of the disease. By examining the clinical features and management challenges of LD in an Apulian cohort, we sought to enhance understanding of this devastating disease and contribute to the development of more effective therapeutic strategies, as well as guide future research directions. Building upon the valuable insights into specific aspects of LD within this cohort from our previous work [3, 4], this manuscript provides a more comprehensive and longitudinal analysis of the entire group, aiming to provide a more complete picture of the disease progression and management. Through a narrative analysis of our clinical experience, we aim to gain a deeper understanding of the lived experience of LD patients and their families in Apulian context, where the disease seems to have a higher prevalence. The contributions of Apulian LD families have been crucial in enhancing both diagnosis and, more importantly, the management of LD. Their experiences with the natural history of the disease have provided valuable insight into its progression, which could guide the identification of therapeutic targets and the development of management strategies for its intermediate and late stages.

## Methods

This retrospective case series study included six unrelated families with LD diagnosed and followed at the Neurology Unit of the Scientific Institute Casa Sollievo della Sofferenza Hospital in San Giovanni Rotondo, Italy, between 2010 and 2024. Comprehensive clinical, video-EEG-polygraphic, laboratory, and metabolic assessments were performed to monitor disease progression (see references 3 and 4 for detailed methods). Inclusion criteria included a confirmed diagnosis of LD based on clinical and genetic findings. Data were collected retrospectively from the medical records of all included patients. Information collected included:

- *Demographic data* age at diagnosis, sex, family history of LD.
- *Clinical presentation* age at onset of symptoms, seizure types, myoclonus severity, cognitive decline, motor deficits (ataxia, dysarthria), and other neurological manifestations.
- *Treatment history* anti-seizure medications (ASMs) (with dosages and duration of treatment), other medications, and any surgical interventions.
- *Disease progression* clinical course of the disease, including the emergence of new symptoms (e.g., dementia, ataxia), functional decline (assessed using functional scales, if applicable), and the development of complications (e.g., aspiration pneumonia, respiratory failure).
- *Outcome data* survival time, cause of death, and end-of-life care (e.g., hospice care, palliative care).

The descriptive statistics for summarizing patient characteristics and clinical features were performed using SPSS (Statistical Package for the Social Sciences). Descriptive statistics were used to summarize patient characteristics and clinical features. The frequency and severity of different seizure types were analyzed.

This study was conducted in accordance with the Declaration of Helsinki and approved by the Institutional Review Board of the Scientific Institute Casa Sollievo della Sofferenza.

## Results

Our experience with LD began fourteen years ago when we observed the first patient in six unrelated families. Genetic analysis revealed EPM2A gene variants in four families and EPM2B gene variants in the remaining two (see Table 1).

**Table 1:** Genetic features of Lafora disease patients

Pt/Sex	Gene	cDNA variation	Protein variation	Type	State	Status
1/F	EPM2A NM_005670.4	c.721C > T	p.(Arg241*)	Nonsense	Homozygous	Affected
2/M	EPM2A NM_005670.4	c.721C > T	p.(Arg241*)	Nonsense	Homozygous	Affected
3/M	EPM2A NM_005670.4	c.721C > T	p.(Arg241*)	Nonsense	Homozygous	Affected
4/M	EPM2A NM_005670.4	c.243_246del c.721C > T	p.(Asp82Argfs*7) p.(Arg241*)	Frameshift Nonsense	Compound Heterozygous	Affected
5a/F	NHLRC1 NM_198586.3	c.992del	p.(Gly331Glufs*3)	Frameshift	Homozygous	Affected
5b/M	NHLRC1 NM_198586.3	c.992del	p.(Gly331Glufs*3)	Frameshift	Homozygous	Affected
6a/M	NHLRC1 NM_198586.3	c.436G > A c.838G > A	p.(Asp146Asn) p.(Glu280Lys)	Missense Missense	Compound Heterozygous	Affected
6b/M	NHLRC1 NM_198586.3	c.436G > A c.838G > A	p.(Asp146Asn) p.(Glu280Lys)	Missense Missense	Compound Heterozygous	Affected

Pt, patient; F, famale; M, male

### Clinical course

Our analysis of the clinical course of LD in our Apulian cohort revealed three distinct electroclinical stages. The initial Presenting Symptoms Stage (early stage) was characterized by the onset of epilepsy, which typically manifested as sporadic tonic–clonic seizures or focal visual seizures. Within a few months to approximately one year following the onset of epilepsy, myoclonic jerks frequently developed. These were often more pronounced upon awakening and predominantly affected the upper body. Electroencephalography (EEG) findings at this stage commonly revealed generalized spike-wave and polyspike-wave discharges, sometimes with an occipital predominance or sporadic focal occipital epileptiform abnormalities. Approximately two years after the onset of epilepsy, patients progressed to the Progressive Neurodegeneration Stage (intermediate stage). This phase was marked by progressive neurological deterioration, including the development of drug-resistant epilepsy, dementia, ataxia, and severe myoclonus. EEG findings during this stage showed a slowing of background activity along with an increased frequency and amplitude of generalized spike-wave and polyspike-wave discharges, often temporally associated with myoclonic jerks. Focal occipital spikes could also be observed during wakefulness and sleep. As the disease advanced, patients entered the Terminal Stage, characterized by a significant decline in functional abilities and overall quality of life, eventually leading to a completely bed-bound state. Seizure emergencies, severe motor impairments, feeding difficulties, and an increased susceptibility to infections became prominent features during this final stage of the disease (see Table 2).

**Table 2:** Clinical features of Lafora disease patients

Pt/Sex	Age early stage onset (y)	Age intermediate stage onset (y)	Age late stage onset (y)	Seizure Type	Status Epilepticus Type	Myoclonus Score	Dementia	Medical Complications	Disease Duration (y)	Conditions at last Follow-up
1/F	13	15	19	Myoclonic, Tonic–Clonic	Myoclonic, Myoclonic-Tonic; NCSE	5	Severe	Dysphagia, aspiration pneumonia, bedsores	10	Mute and bedridden, with PEG and tracheostomy
2/M	13	16	22	Myoclonic, Tonic–Clonic	Myoclonic, Myoclonic-Tonic; Focal Motor; Tonic; NCSE	5	Severe	Dysphagia, aspiration pneumonia	12	Mute and bedridden, with PEG. Deathat age 25 from pneumonia
3/M	10	13	18	Myoclonic, Tonic–Clonic	Myoclonic; Myoclonic-Tonic	5	Severe	Dysphagia, aspiration pneumonia, bedsores	16	Mute and bedridden, with PEG. Death at age 26 from pneumonia
4/M	12	16	18	Myoclonic, Tonic–Clonic	Myoclonic; Myoclonic-Tonic	5	Moderate	aspiration pneumonia	6	Severe ataxia, limited interaction. Death at age 18 from pneumonia
5a/F	13	15	19	Tonic–Clonic	–	5	Severe	Dysphagia, aspiration pneumonia	11	Mute and bedridden, with PEG. Death at age 24 from SUDEP
5b/M	14	17	20	Myoclonic Tonic–Clonic	Tonic–Clonic	4	Moderate	Dysphagia, aspiration pneumonia	6	Moderate ataxia, limited interaction. Death at age 20 from sepsis
6a/M	13	16	–	Myoclonic Tonic–Clonic	Myoclonic; Myoclonic-Tonic	3	Moderate	Dysphagia, aspiration pneumonia	9	Moderate ataxia and interaction
6b/M	14	18	–	Myoclonic Tonic–Clonic	–	2	Mild	-	6	Mild ataxia, good interaction

Pt, patient; F, famale; M, male; LD, Lafora disease; y,years; F, female; M, male. PEG: percutaneous endoscopic gastrostomy; NCSE: Nonconvulsive SE with coma

Myoclonus Score: 0 no myoclonus; 1 minor myoclonus, no interference with daily living; 2 mild myoclonus, interference with fine movements and/or speech, no interference with walking; 3 moderate myoclonus, patient still able to walk without support; 4 moderate to severe myoclonus, patient able to stand, unable to walk without support; 5 severe myoclonus, patient wheelchair-bound or bedridden*. (Magaudda A, Gelisse P, Genton P., Epilepsia 2004; 45:678–681)*

### Management challenges

**Fig. 1:**
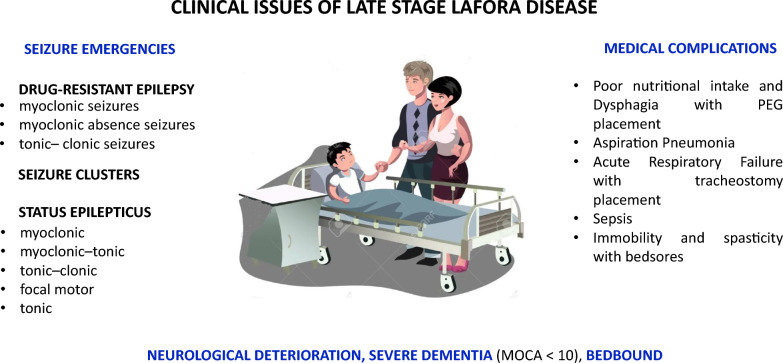
Seizure Emergencies and Medical Complications of Late Stage: seizure emergencies associated with multiple medical complications were the two main clinical and critical aspects

The management of LD presented several challenges, particularly in the later stages (as summarized in Fig. 1). Regarding Seizure Management, drug-resistant seizures became increasingly frequent as the disease progressed, often necessitating polytherapy with a combination of three to four ASMs. Myoclonic status epilepticus was identified as a frequent and particularly challenging seizure type to manage. In our experience, intravenous benzodiazepines and levetiracetam were often effective for the initial control of seizures, while intravenous phenytoin demonstrated efficacy in controlling motor non-exclusively myoclonic status epilepticus. The Medical Complications observed significantly impacted patient outcomes in the late stages of the disease. These included aspiration pneumonia, respiratory failure, and the development of pressure ulcers. Furthermore, poor nutritional intake and dysphagia frequently necessitated the implementation of feeding support through nasogastric tubes or percutaneous endoscopic gastrostomy (PEG) tubes.

### Multidisciplinary care

**Fig. 2:**
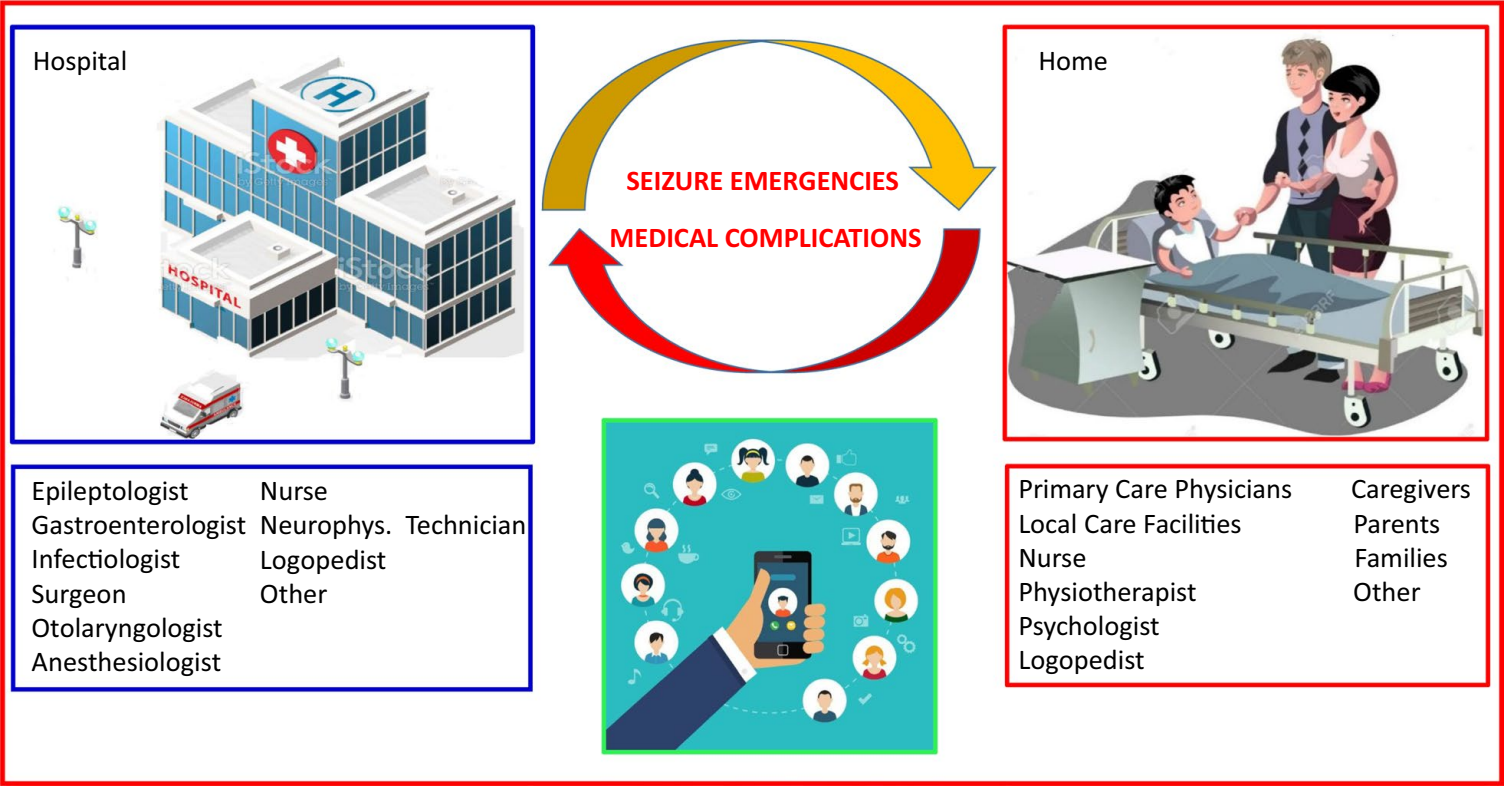
Management of Late Stage: LD patients were kept at home and followed by Epilepsy team, the medical and the nursing team working in the hospital and in the home setting, and the local caregiving structure by means of a remote and an Internet connection. Hospitalization was limited to seizure and medical emergencies not treatable in the home setting

A Multidisciplinary approach involving neurologists, epileptologists, nurses, physical therapists, and other healthcare professionals was crucial for addressing the complex and evolving needs of patients in the late stages of LD (as illustrated in Fig. 2). In cases where home-based care included the administration of intravenous medication for acute seizure emergencies, this was conducted under the strict supervision of our specialized epilepsy team. Furthermore, home-based care, with the essential support of healthcare professionals and dedicated caregivers, proved vital for optimizing patient management and minimizing the necessity for frequent hospitalizations.

## Discussion

Previous studies have described the clinical course of LD, including the emergence of drug-resistant epilepsy, progressive neurodegeneration, and ultimately, death [1, 3, 5–11]. However, limited data exist on the long-term management of the disease, particularly in specific geographic regions such as Apulia, where a higher prevalence of LD has been reported [4, 12]. Our findings provide valuable insights into the clinical course of LD and may serve to inform future research and clinical management strategies (see Table 3). LD, like other rare and ultra-rare diseases, is often late or misdiagnosed as juvenile myoclonic epilepsy (JME), especially in the absence of a family history [4]. For a neurodegenerative and progressive disease like LD, where time is critical, early diagnosis is crucial to initiate timely interventions, such as repurposing existing drugs like metformin and exploring novel therapeutic strategies [13]. A multidisciplinary approach is essential for addressing the diverse clinical manifestations of LD. Through comprehensive clinical evaluations, including prolonged video-EEG recordings, we identified distinct electro-clinical features in our cohort that strongly suggested an LD diagnosis [4]: (1) the progression from tonic–clonic and focal visual seizures to bilateral tonic–clonic seizures; (2) later myoclonic jerks, not exclusively upon awakening; (3) EEG background slowing with superimposed sporadic, diffuse epileptiform abnormalities, often with a maximum discharge over the occipital region. With the onset of additional symptoms (cognitive decline, ataxia), the implementation of tailored rehabilitation programs, including motor, cognitive, and swallowing therapy, is crucial to manage associated symptoms such as motor and cognitive decline and dysphagia. Similarly, when drug resistance to epilepsy and continuous myoclonus develop, a polytherapy approach using a limited number of ASMs with anti-myoclonic effects should be prioritezed to ensure better tolerability. This approach may necessitate a personalized therapy to optimize outcomes [14]. The search for new electro-clinical and metabolic biomarkers useful for early diagnosis, for assessing disease progression, and for being used as indicators of response to new or repurposed drugs represents the current and future challenge [13]. Seizure emergencies, including frequent drug-resistant seizures, seizure clusters, and refractory and super-refractory status epilepticus (SE), along with multiple medical complications, were the two main clinical and critical aspects of the late stage in our cohort (Fig. 1). Notably, the occurrence of refractory/super-refractory SE and/or aspiration pneumonia, requiring percutaneous endoscopic gastrostomy (PEG) or tracheostomy placement, marked the transition from the intermediate to the late stage in all patients. Therefore, a comprehensive and coordinated management approach to both seizure emergencies and medical complications is crucial for optimal patient outcomes in this final phase of the disease. Six to ten years after the epilepsy onset, drug-resistant seizures occur monthly or several times per month, often in the form of seizure clusters. Moreover, seizure chronic treatment evolves to polytherapy, typically involving a combination of three to four drugs. Newer ASMs, including second and third generation ASMs, have notably improved tolerability, though they have not significatley impacted the response or overall long-term LD progression [1, 2, 14]. Nervertheless, the most important seizure emergency is represented by SE, with its most common form being SE with prominent motor symptoms. Myoclonic SE is a very frequent type of SE and has been characterized by repetitive, multifocal, both rhythmic and arrhythmic myoclonic jerks, associated with diffuse, multifocal, and faster discharges of spike and wave/polispike and wave. Intravenous benzodiazepines, often followed by intravenous levetiracetam, usually resolve SE, especially if given at the onset. However, when particularly massive, myoclonic jerks were intermixed with the increase of muscle tonus and breathing difficulties, establishing a myoclonic-tonic SE. Non-exclusively myoclonic subtypes, such as myoclonit-tonic seizures, either had no response or only a limited and transient response to benzodiazepines and newer ASMs. In contrast, intravenous phenytoin was effective in controlling motor non-exclusively myoclonic SE in 75% of cases, thereby prevending access to the ICU and avoiding the transition to refractory to super-refractory SE. Therefore, polygraphic studies are fundamental in the last stage of LD:

- to avoid misdiagnosis, as the electro-clinical features of non-exclusively myoclonic SE can sometimes resemble those of myoclonic SE;
- to accurately diagnose the specific subtype of status epilepticus;
- to guide the selection of appropriate ASMs for seizure control.

**Table 3:** xbonl

Therapeutic Interventions	Lafora disease	Biomarker discovery
*Symptomatic treatment* Anti-seizure medications, anti-myoclonic drugs	*Presenting symptom stage* •Clinical Manifestations: Seizures (tonic–clonic, focal visual I, myoclonus •Diagnostic challenges: Misdiagnosis as JME •Key to Earty Diagnosis: identification of specific etectro-clifMcal features	Early diagnosis bio markers; Electro-clinical features
*Symptomatic treatment* Anti-seizure mediations, anti-myoclonic drugs *Drug repurposing* Metformin, other *Novel Therapies* Research and development	*Progressive neurodegeneration stage* • Clincal Manifestations: Cognitive decline, ataxia. dysphagia, drug resistant epilepsy, myoclonus •Management: Multidisciplinary approach, tailored rehabilitation, polytherapy	*Disease progression biomarker:* EEG, Metabolic, Neuroimaging markers Therapeutic Response Bio markers: Clinical measures, biological markers
*Symptomatic Treatment* Anti-seizure medications, anti-myoclonic drugs	*Late Stage* • Clinical Manifestations: Cognitive decline, ataxia, dysphagia, drug resistant epilepsy myoclonus *Management*: Multidisciplinary approach, tailored rehabilitation, polytherapy	*Disease Progression Biomarkers*: EEG, Metabolic, Neuroimaging markers

Based on our experience, we propose the use of intravenous benzodiazepines, followed by intravenous levetiracetam for myoclonic SE, and intravenous benzodiazepines followed by intravenous phenytoin for motor non-exclusively myoclonic SE.

Moreover, after the acute phase in the hospital setting, clusters or motor SE which reoccurred in the home setting were treated with intravenous benzodiazepines for a short period, and particularly with intravenous phenytoin, which provided a rapid and complete control, preventing the need for further hospitalizations (Fig. 2).

The management of medical complications is as important as seizure emergency diagnosis and treatment. In fact, our patients experienced poor nutritional intake and dysphagia with the administration of food and fluids via a nasogastric tube and, subsequently, via a PEG. Other common complications included aspiration pneumonia, acute respiratory failure with tracheostomy placement, sepsis, immobility, and spasticity, which resulted in a higher rate of pressure ulcers (Fig. 1).

Depending on the local availability of devices and parents’ and caregivers’ skills, LD patients were kept at home and followed by our epilepsy team, the medical and the nursing team working in the hospital and in the home setting, and the local caregiving structure. Comunication was mainatined by means of phone calls, secure messaging smartphone applications and even videocalls if needed, in other words by means of a remote and an Internet connection. Hospitalization was reserved to seizure and medical emergencies not treatable in the home setting (Fig. 2).

Therefore, our epilepsy center coordinated a multidisciplinary medical and nursing team working both in the hospital and in the home setting. The goal of this team was to prevent and manage multiple complications, while also providing remote assistance to primary care physicians and local care facilities in the home setting, in order to avoid repetitive hospitalizations. Physical therapy was also continued in this late stage to maintain a good overall muscular condition, treat spasticity, and prevent medical complications. Finally, psychological and social support was very important, especially for LD parents and caregivers, which should receive professional and constant support.

## Conclusion and future perspectives

Although limited by its retrospective design and the relatively small sample size, this study provides valuable insights into the clinical course and management challenges of LD in a cohort of patients from the Apulia region of Southern Italy. Our findings highlight the critical role of early diagnosis in enabling timely interventions, as well as the importance of a multidisciplinary approach to address the complex needs of these patients. The late stages of LD are characterized by significant challenges, including frequent seizure emergencies, refractory status epilepticus, and the emergence of severe medical complications. Effective management in these late stages requires a robust multidisciplinary approach involving neurologists, epileptologists, nurses, physical therapists, dieticians, and other healthcare professionals. Home-based care, with strong support from healthcare professionals and caregivers, is essential for optimizing patient management and minimizing the need for frequent hospitalizations. It is important to note that when intravenous medications were administered at home for acute seizure management, this was always performed under the direct and continuous supervision of our specialized epilepsy team.

Managing LD is akin to a complex basketball “game”. Early diagnosis and intervention are crucial for establishing an early advantage. However, even when facing challenges in the later stages, a well-coordinated team effort, with each player (clinician, patient, family, researcher) playing a vital role, can significantly improve the outcome. In fact, this “game” is constantly evolving, and the team must continually adapt its strategies to address the changing needs of the patient. Nevertheless, we often find ourselves in the second half of this game, having missed the early opportunities due to delayed or misdiagnosis. Our target now is to prolong the match and potentially reach a more favorable outcome through an effective multidisciplinary approach, while eagerly awaiting the development of new therapeutic strategies.

Successful late-stage LD management necessitates a robust multidisciplinary network of professionals working both in the home setting and in hospitals, coordinated by specialized epilepsy teams, with active involvement from parents and caregivers. In this “game”, the role of the epileptologists is pivotal. They act as the “playmaker”, orchestrating the efforts of other players, including physicians, parents, caregivers, and researchers, within this multidisciplinary team. Furthermore, the epileptologist plays a crucial role in ensuring the effective management of seizure emergencies and overseeing the complex interplay of medical complications. This requires not only clinical expertise but also emotional and psychological support for patients and their families, particularly during the challenging late stages of the disease. As Michael Jordan famously stated, achieving championship success requires not just individual talent but also intelligence, heart, and unwavering teamwork. Similarly, successful LD management demands a multidisciplinary approach driven by collaboration, empathy, and a shared commitment to improving the quality of life for both patients and their families. Our findings emphasize the need for continued research on targeted therapies and supportive care strategies for LD patients. Ongoing research into the pathophysiology of LD is essential for the identification of novel biomarkers for early diagnosis and disease progression as well as for the development of disease-modifying therapies. In this context, repurposing existing drugs throug computational and pharmacological approaches can serve as a strategy to accelerate the identification of effective therapies. Medicinal chemists and pharmacologists play a crucial role in achiving this goal, making them others players in the team to defeat LD.

Beyond these clinical and scientific aspects, our long-term involvement with the Apulian families has underscored the profound daily impact of LD on their lives and the critical need for comprehensive support systems.

In conclusion, this study highlights the significant clinical burden of LD and underscores the importance of early diagnosis and a multidisciplinary approach to management, particularly in the challenging late stages of the disease. Home-based care, supported by healthcare professionals and caregivers, plays a crucial role in optimizing patient well-being. Regarding the observed higher prevalence of LD in the Apulia region, we emphasize the need for future research to investigate potential genetic or environmental factors contributing to this trend, as such insight could guide targeted interventions and public health strategies within this population. Continued research into novel therapeutic strategies remains paramount for this devastating condition.

## Data Availability

Not applicable.
